# *In Vivo* Competitions between Fibrobacter succinogenes, Ruminococcus flavefaciens, and Ruminoccus albus in a Gnotobiotic Sheep Model Revealed by Multi-Omic Analyses

**DOI:** 10.1128/mBio.03533-20

**Published:** 2021-03-03

**Authors:** Carl J. Yeoman, Christopher J. Fields, Pascale Lepercq, Philippe Ruiz, Evelyne Forano, Bryan A. White, Pascale Mosoni

**Affiliations:** a Department of Animal and Range Sciences, Montana State University, Bozeman, Montana, USA; b Biotechnology Center, University of Illinois at Urbana-Champaign, Urbana, Illinois, USA; c TBI, Université de Toulouse, CNRS, INRAE, INSA, Toulouse, France; d Université Clermont Auvergne, INRAE, UMR 454 MEDIS, Clermont-Ferrand, France; e Carl R. Woese Institute for Genomic Biology, University of Illinois at Urbana-Champaign, Urbana, Illinois, USA; f Department of Animal Sciences, University of Illinois, Urbana, Illinois, USA

**Keywords:** pili IV, CAZymes, cellulose degradation, gnotobiotic animal model, outer membrane vesicles, rumen

## Abstract

Fibrobacter succinogenes, Ruminococcus albus, and Ruminococcus flavefaciens are the three predominant cellulolytic bacterial species found in the rumen. *In vitro* studies have shown that these species compete for adherence to, and growth upon, cellulosic biomass. Yet their molecular interactions *in vivo* have not heretofore been examined. Gnotobiotically raised lambs harboring a 17-h-old immature microbiota devoid of culturable cellulolytic bacteria and methanogens were inoculated first with F. succinogenes S85 and *Methanobrevibacter* sp. strain 87.7, and 5 months later, the lambs were inoculated with R. albus 8 and R. flavefaciens FD-1. Longitudinal samples were collected and profiled for population dynamics, gene expression, fibrolytic enzyme activity, *in sacco* fibrolysis, and metabolite profiling. Quantitative PCR, metagenome and metatranscriptome data show that *F. succinogenes* establishes at high levels initially but is gradually outcompeted following the introduction of the ruminococci. This shift resulted in an increase in carboxymethyl cellulase (CMCase) and xylanase activities but not in greater fibrolysis, suggesting that *F. succinogenes* and ruminococci deploy different but equally effective means to degrade plant cell walls. Expression profiles showed that *F. succinogenes* relied upon outer membrane vesicles and a diverse repertoire of CAZymes, while R. albus and *R. flavefaciens* preferred type IV pili and either CBM37-harboring or cellulosomal carbohydrate-active enzymes (CAZymes), respectively. The changes in cellulolytics also affected the rumen metabolome, including an increase in acetate and butyrate at the expense of propionate. In conclusion, this study provides the first demonstration of *in vivo* competition between the three predominant cellulolytic bacteria and provides insight on the influence of these ecological interactions on rumen fibrolytic function and metabolomic response.

## Abstract (precis)

Ruminant animals, including cattle and sheep, depend on their rumen microbiota to digest plant biomass and convert it into absorbable energy. Considering that the extent of meat and milk production depends on the efficiency of the microbiota to deconstruct plant cell walls, the functionality of predominant rumen cellulolytic bacteria, Fibrobacter succinogenes, Ruminococcus albus, and Ruminococcus flavefaciens, has been extensively studied *in vitro* to obtain a better knowledge of how they operate to hydrolyze polysaccharides and ultimately find ways to enhance animal production.

## INTRODUCTION

Cellulose typically represents ∼40% of plant biomass (1) but is not endogenously degradable by mammalian species, including ruminants, due to a lack of an endogenously encoded ability to hydrolyze this important carbohydrate resource. Mammals are instead dependent on members of their gastrointestinal tract microbiota for providing this capability (2). Ruminant animals, including cattle and sheep, harbor a core rumen microbiome (3), including a limited number of taxa known to specialize in the deconstruction of cellulose (4, 5). The three best described and cultivable cellulolytic bacteria, Fibrobacter succinogenes, Ruminococcus albus, and Ruminococcus flavefaciens, each employ unique methods of fulfilling this function. R. flavefaciens facilitates cellulose deconstruction by virtue of a cellulosome (6); R. albus instead appears to utilize noncellulosomal, but coalescing membrane-attached enzymes (7), while the organization of the cellulolytic system of F. succinogenes remains enigmatic, with outer membrane vesicles (OMVs) possibly involved (8, 9). Each of these species is frequently detected among a variety of adult ruminants throughout the world and often cooccur in the rumen (3, 4). Previous *in vitro* studies have provided evidence of competition among these cellulolytic species for adhesion to plant biomass and for growth on cellulose substrates (10–12). However, little is known about their symbiotic relationships *in vivo* or the antagonistic or synergistic impact of their cooccurrence on fibrolysis. We sought to determine how the three species interact molecularly *in vivo* within a previously described and tractable gnotobiotic sheep model (13, 14) and to evaluate how variations in their composition affect ruminal fibrolysis and metabolism alongside a methanogenic hydrogenotroph.

## RESULTS

### Fibrobacter succinogenes is outcompeted following inoculation of ruminococci.

**FIG 1:**
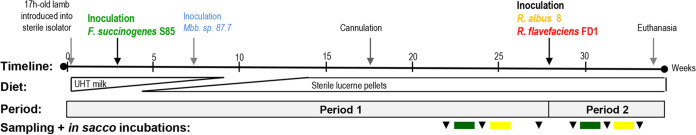
Overview of animal trial. The lambs were initially fed sterilized cow milk (until weaning at week 14) and then sterilized lucerne hay pellets. They were inoculated with *F. succinogenes* S85 (week 3), *Methanobrevibacter* (*Mbb*.) sp. 87.7 (week 7), and R. albus 8 and *R. flavefaciens* FD1 (week 28). Periods 1 and 2 correspond to pre- and postinoculation of the ruminococci, respectively. Sampling of rumen contents during both periods are indicated by black triangles. *In sacco* incubations of lucerne and wheat straw cell walls are indicated by green and yellow rectangles, respectively.

**FIG 2:**
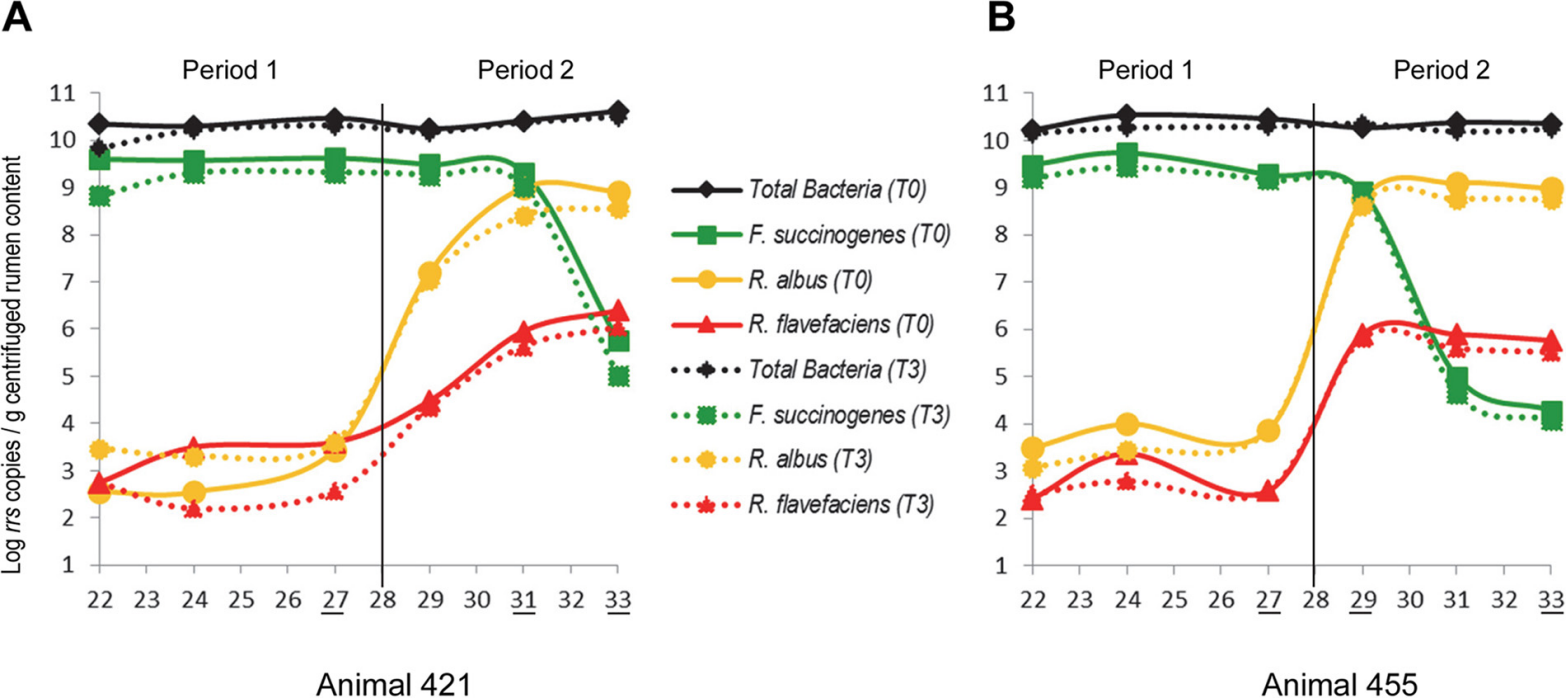
Dynamics of total bacteria and *F. succinogenes*, R. albus, and *R. flavefaciens*. The numbers of 16S copies/gram of rumen material evaluated by quantitative PCR in rumen contents pre- and postinoculation of the ruminococci (at week 28) are shown.

Fibrobacter succinogenes S85 (week 3), *Methanobrevibacter* sp. strain 87.7 (week 7), Ruminococcus albus 8, and *R. flavefaciens* FD1 (week 28) were sequentially inoculated into two gnotobiotic lambs that were raised aseptically from birth to 37 weeks (Fig. 1). Microbial composition dynamics were monitored from weeks 22 to 33 using quantitative PCR (qPCR) (Fig. 2) and most probable number (MPN) enumeration of total cultivable and total cultivable cellulolytic bacteria (see Fig. S1 in the supplemental material). Using qPCR, the total number of bacteria observed throughout the sampling period showed little variation at 10^10.3 ± 0.16^ 16S rDNA copies/g of rumen content (Fig. 2). These numbers did not vary between the two animals (*P* = 0.5) and were consistent with those measured by MPN (10^10.2 ± 0.4^ cells/ml) (Fig. S1A and S1B). qPCR data indicated that *F. succinogenes* was initially dominant, being enumerated at 10^9.4 ± 0.25^ 16S rDNA copies/g prior to inoculation of the two *Ruminococcus* strains. Following inoculation with the two *Ruminococcus* strains, *F. succinogenes* was gradually outcompeted in both animals. By week 33, R. albus and *R. flavefaciens* were enumerated at 10^8.8 ± 0.2^ and 10^5.9 ± 0.4^ 16S rDNA copies/g, respectively, while *F. succinogenes* was enumerated at 10^4.7 ± 0.8^ 16S rDNA copies/g (Fig. 2). *Methanobrevibacter* sp. 87.7 was enumerated at 10^5.8 ± 0.9^ 16S rDNA copies/g, and total cultivable methanogens were enumerated by MPN at 10^6.2 ± 1.01^ cells/ml throughout the sampling period and exhibited noticeable fluctuation over the first 3 weeks following inoculation of the *Ruminococcus* spp. (Fig. S1C). Consistent with both qPCR and MPN data, most metagenomic and metatranscriptomic reads aligned to the *F. succinogenes* S85 reference genome at week 27 with only low to modest alignment to the *Methanobrevibacter* sp. 87.7 and the two *Ruminococcus* genomes (Fig. S2A). *F. succinogenes* was also well represented in metagenomic, but not metatranscriptomic, data from weeks 29 and 31 and obtained almost no coverage by week 33. This transition, was also seen in the increasing alignment of these data to the R. albus and *R. flavefaciens* genomes between weeks 29 and 33 (Fig. S2A).

10.1128/mBio.03533-20.4 FIG S1 (A and B) Dynamics of cultivable total bacteria and cellulolytic bacteria in rumen contents withdrawn before morning feeding (A) and 3 h after feeding (B). (C) Dynamics of methanogens evaluated by quantitative PCR in rumen contents pre- and postinoculation of the ruminococci (at week 28) at T0 (prefeeding) and T3 (postfeeding). (D) pH monitoring of rumen contents during the course of the animal trial. Download FIG S1, PDF file, 0.1 MB. Copyright © 2021 Yeoman et al. 2021 Yeoman et al. https://creativecommons.org/licenses/by/4.0/ This content is distributed under the terms of the Creative Commons Attribution 4.0 International license.

10.1128/mBio.03533-20.5 FIG S2 (A) Comparative genomic coverage. Reads for metagenomic and metatranscriptomic samples were preprocessed to remove adapters and low-quality data using Trimmomatic v.0.33. Reads were then aligned to the four reference genomes using bwa v0.7.15. Each row represents read depth for the four reference strains. Genomic coverage (blue) and transcriptome coverage (red) (depth is represented at fixed log scale for all four strains) are shown. (B) CAZy-related expression analysis for reference samples. Expression counts were generated for all four genomes from the metatranscriptomic reads using featureCounts v. 1.5.2. All genes for the four genomes were then assessed for CAZy-related protein motifs using HMMER v3.1b and the Release 5.0 models attained from dbCAN; only genes with one or more significant hits were retained in this analysis based on the parameters suggested by the dbCAN curators. Gene expression was scaled to standard deviation from the mean for scaling purposes. The species are indicated as follows: *F. succinogenes* S85 in green, *Methanobrevibacter* sp. 87-7 in blue, R. albus 8 in yellow, and *R. flavefaciens* FD-1 in red. Download FIG S2, PDF file, 0.2 MB. Copyright © 2021 Yeoman et al. 2021 Yeoman et al. https://creativecommons.org/licenses/by/4.0/ This content is distributed under the terms of the Creative Commons Attribution 4.0 International license.

### Gene expression.

**FIG 3:**
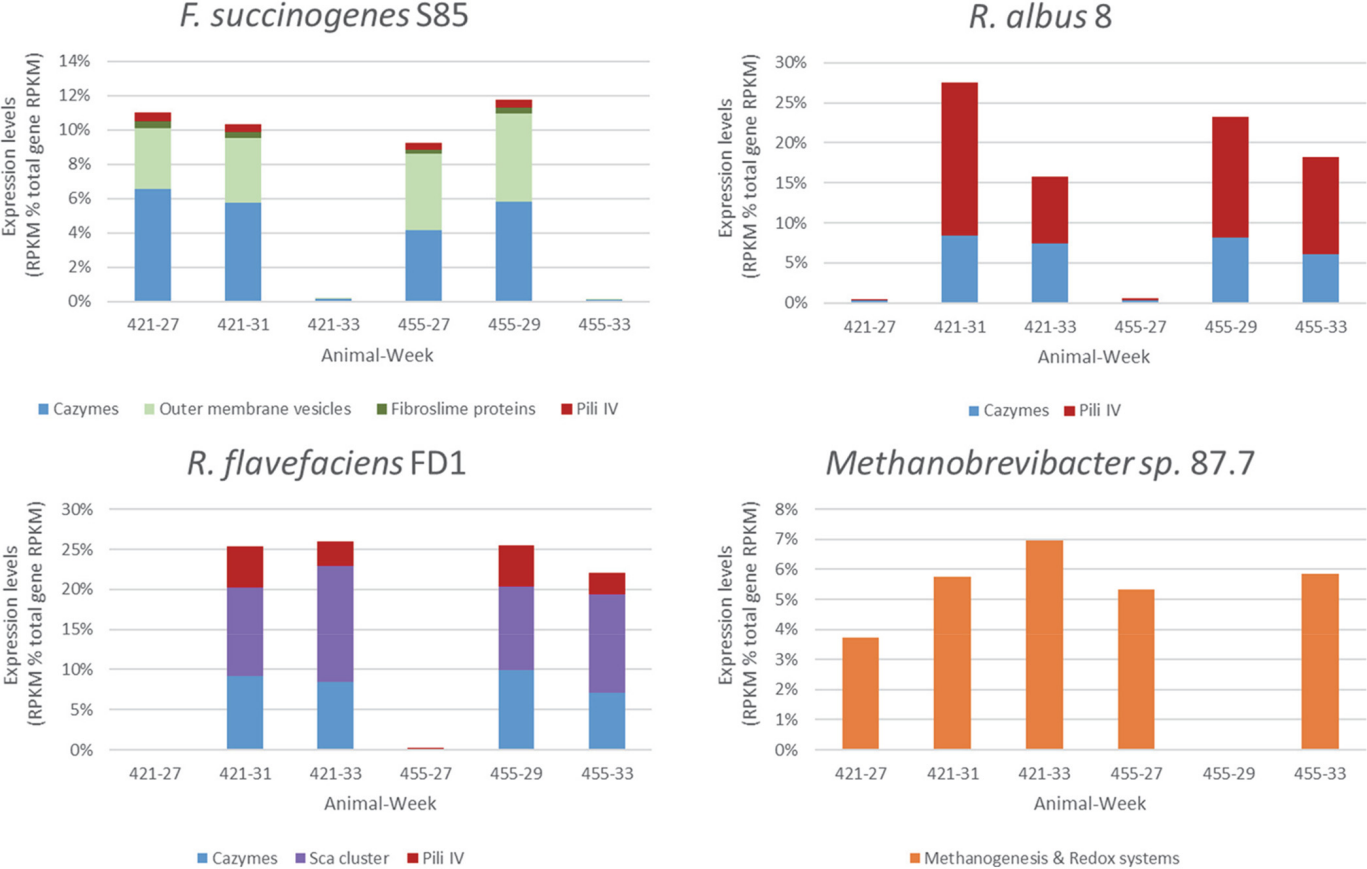
Major functional or structural genes expressed by the four inoculated strains *in vivo.* Relevant functional or structural genes expressed by *F. succinogenes* S85, R. albus 8, *R. flavefaciens* FD-1, and *Methanobrevibacter* 87.7 are given in Table S1 in the supplemental material, in addition to the calculations of the percentages of each group of expressed genes within each genome. These percentages were used to generate the four histograms presented here.

**FIG 4:**
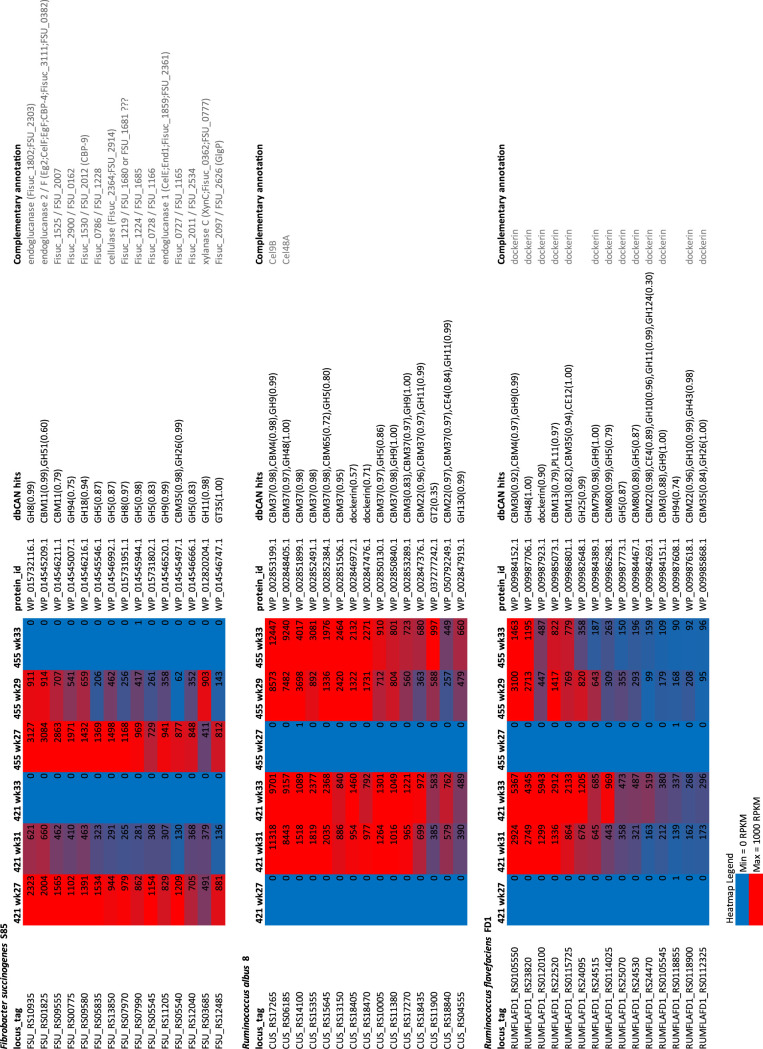
Top 15 CAZy gene expression levels by the three inoculated cellulolytic strains *in vivo*. The 15 most abundant CAZyme transcripts of each cellulolytic strain were retrieved from Table S1. CAZyme transcripts of *F. succinogenes* S85, R. albus 8, and *R. flavefaciens* FD-1 were sorted in Table S1 from the highest to lowest expression counts (expressed in RPKM) using the sum of counts of each transcript in the two animals (421 and 455) at the three sampling times. For each gene, the encoded protein identifier (ID) is given along with its modular composition (dbCAN hits) and complementary annotation provided on the CAZy site (http://www.cazy.org/) or determined by manual annotation using BLASTP on NCBI (https://blast.ncbi.nlm.nih.gov/Blast.cgi).

**FIG 5:**
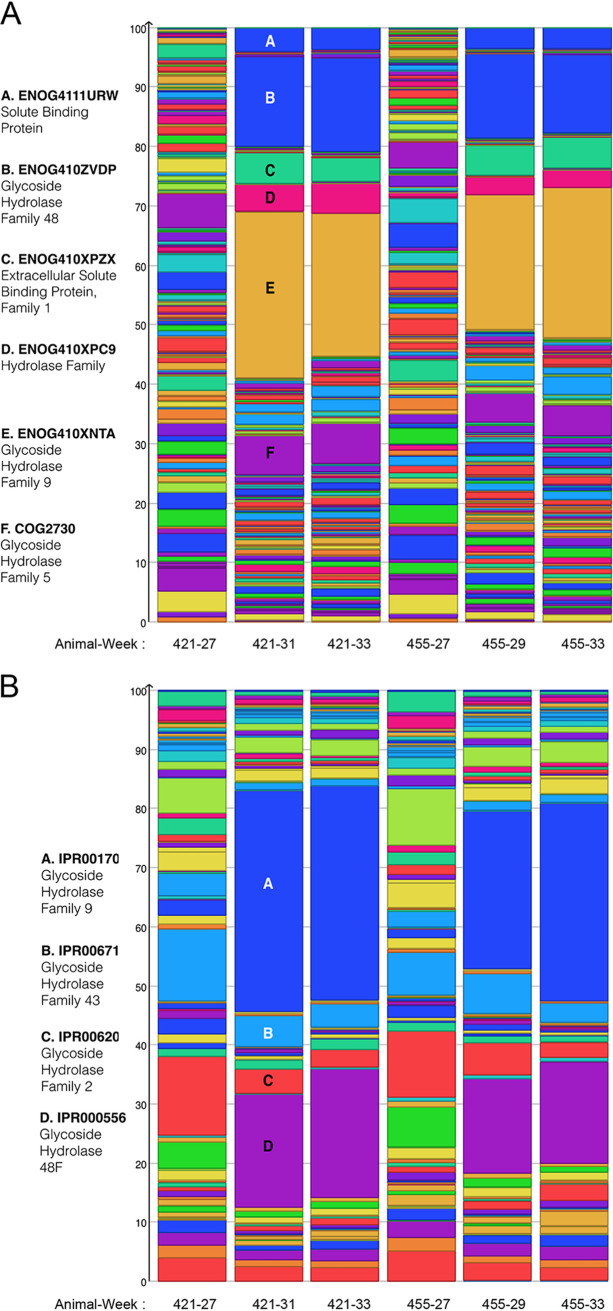
Expression profiles for eggnog/COG (clusters of orthologous groups) (A) and Interpro2GO categories (B). Counts were derived from functional assignments to the full metatranscriptome read data. (A) Overall counts for assignments to functions related to carbohydrate transport and metabolism (family G) were then determined using MEGAN, scaling to the overall percentage of each specific functional category, with the top six eggnog categories described. (B) Overall counts for assignments to functions related to carbohydrate metabolism (GO:0005975, carbohydrate metabolic process) were then determined using MEGAN, scaling to the overall percentage of each specific functional category, with the top four InterPro assignments shown.

Carbohydrate-active enzymes (CAZymes) and accessory systems putatively involved in bacterial adhesion or polysaccharide utilization represented 11%, 21%, and 25% of the total transcriptomes of *F. succinogenes*, R. albus, and *R. flavefaciens*, respectively (Fig. 3), while *Methanobrevibacter* sp. 87.7 expressed mostly genes involved in methanogenesis and redox systems (on average 5.5% of total gene expression) (Fig. 3). The expression of CAZymes by each cellulolytic species reflected each bacterium’s relative abundance in each sample (Fig. S2A and S2B). Although the inoculated cellulolytic strains each encoded approximately 100 CAZyme genes (see http://www.cazy.org/), only a small subset of those genes were expressed at >1% of their total CAZy gene expression (Fig. 4; see Table S1 in the supplemental material). The 15 most highly expressed CAZyme genes from each of the three cellulolytic strains across all time points were genes targeting cellulose (glycoside hydrolase 5 [GH5], GH8, GH9, GH48, and GH51), hemicelluloses (GH10, GH11, GH26, GH43, and carbohydrate esterase 4 [CE4]) and pectins (polysaccharide lyase 11 [PL11] and CE12). Notably, each of R. albus’ and *R. flavefaciens*’ 15 most expressed CAZyme transcripts encoded proteins harboring either a family 37 carbohydrate-binding module or a dockerin, respectively (Fig. 4). A more diverse carbohydrate transport and metabolism gene expression profile was measured when the ecosystem harbored high numbers of *F. succinogenes* S85 (period 1) compared to that observed following the introduction of the ruminococci (period 2) (Fig. 5). R. albus and *R. flavefaciens* were observed to most abundantly express GH9 and GH48 family mRNAs and their inoculation resulted in a dramatic increase in the level of these transcripts that corresponded to decreases in GH2 and GH43 mRNAs (Fig. 5). In addition to CAZymes, *F. succinogenes* exhibited high expression of genes predicted to be involved in the production of extracellular structures, including outer membrane vesicles (OMVs) (4% of total expression). R. albus 8 expressed type IV pilus genes (14% of total gene expression), and *R. flavefaciens* FD1 expressed both cellulosomal and type IV pilus genes (12 and 4% of total expression, respectively) (Table S1). Fibro-slime domain containing genes mostly exhibited only low to moderate expression by *F. succinogenes* (0.35% of total gene expression), the exception being FSU_RS11870 (FSU_2502) that was expressed at ∼1,000 reads per kilobase per million (RPKM) initially prior to the introduction of the ruminococci (Table S1).

10.1128/mBio.03533-20.2 TABLE S1 Transcriptomic RPKM data of *F. succinogenes* S85, R. albus 8, and *R. flavefaciens* FD-1 CAZyme genes (A) and genes encoding accessory systems potentially involved in bacterial adhesion or polysaccharide utilization (B) according to animal (421 and 455) and sampling time. Download Table S1, XLSX file, 0.3 MB. Copyright © 2021 Yeoman et al. 2021 Yeoman et al. https://creativecommons.org/licenses/by/4.0/ This content is distributed under the terms of the Creative Commons Attribution 4.0 International license.

### Adhesion to plant biomass.

Using qPCR, we further sought to determine any preferential binding of the three inoculated cellulolytic organisms to plant biomass. The distribution of the three species adherent to cannula-inserted *in sacco* samples of either lucerne (alfalfa) or wheat straw reflected their measured abundances in the rumen content with no obvious preference of each cellulolytic species to one or other substrate (Fig. S3). Consistently, scanning electron microscopy (SEM) analyses of both substrates revealed high densities of adherent rod morphotypes consistent with *F. succinogenes*’ cell morphology (Fig. S4) prior to the inoculation of the two cellulolytic ruminococci. After inoculation of R. albus and *R. flavefaciens*, adherent cocci were observed consistent with the *Ruminococcus* cell morphology (Fig. S4). *F. succinogenes-*like morphotypes remained evident in these later samples although they were never observed alongside *Ruminococcus*-like morphotypes, suggesting spatial partitioning.

10.1128/mBio.03533-20.6 FIG S3 Quantitative determination of adhering microbes to two different plant materials. Total bacteria (black bars), *F. succinogenes* (bars filled with squares), R. albus (bars filled with points), and *R. flavefaciens* (bars filled with stripes) were quantified by qPCR after *in sacco* incubation of lucerne (animal 421 [A] and animal 455 [B]) and wheat straw cell walls (animal 421 [C] and animal 455 [D]). *In sacco* incubations were done in period 1 (weeks 23 and 25) and in period 2 (weeks 30 and 32). Download FIG S3, PDF file, 0.06 MB. Copyright © 2021 Yeoman et al. 2021 Yeoman et al. https://creativecommons.org/licenses/by/4.0/ This content is distributed under the terms of the Creative Commons Attribution 4.0 International license.

10.1128/mBio.03533-20.7 FIG S4 Scanning electron microscopy of adhering microbes to two plant materials. Observations were made after 24-h *in sacco* incubation of the plant materials in the rumen during period 1 (A, lucerne, week 23, animal 455; B, wheat straw, week 25, animal 421) or during period 2 (C, lucerne, week 30, animal 421; D, wheat straw, week 30, animal 421; E, wheat straw, week 32, animal 455). Bar = 10 μm. Download FIG S4, PDF file, 0.4 MB. Copyright © 2021 Yeoman et al. 2021 Yeoman et al. https://creativecommons.org/licenses/by/4.0/ This content is distributed under the terms of the Creative Commons Attribution 4.0 International license.

### Fibrolytic activity.

**FIG 6:**
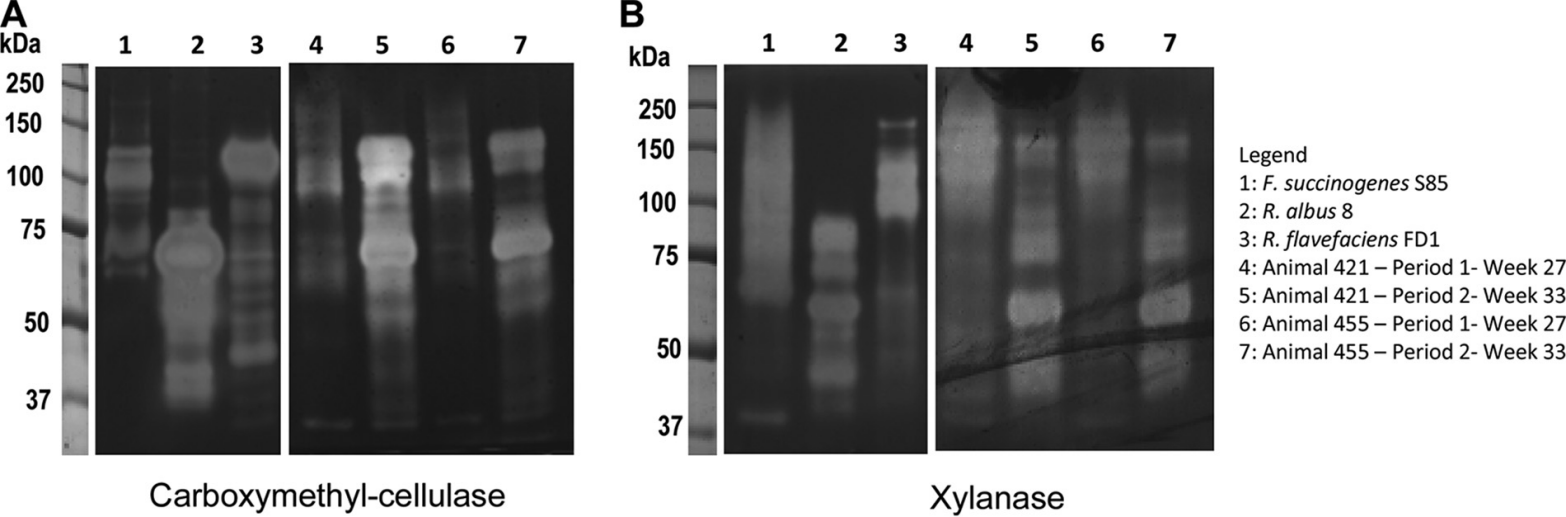
Zymogram profiles of carboxymethycellulases (A) and xylanases (B). Zymograms showing active enzyme profiles of pure cultures and present in protein extracts from the sheep rumen contents at the end of period 1 at week 27 (lane 4, animal 421; lane 6, animal 455) and of period 2 at week 33 (lane 5, animal 421; lane 7, animal 455). Zymogram profiles of protein extracts from *F. succinogenes* S85 (lane 1), R. albus 8 (lane 2), and *R. flavefaciens* (lane 3) after 48-h *in vitro* incubation on filter paper strips are presented for comparison.

Zymogram profiles revealed carboxymethyl cellulase (CMCase) and xylanase enzyme profiles to be consistent with those of a pure culture of *F. succinogenes* initially at week 27 but to be more similar to pure R. albus and *R. flavefaciens* culture profiles at week 33 (Fig. 6). Total rumen CMCase and xylanase specific activity increased 2.6-fold during this same period (Table 1). However, increases in these fibrolytic activities were not reflected by *in sacco* experiments, where no significant increase was seen in the rates of dry matter (DM), neutral detergent fiber (NDF), or acid detergent fiber (ADF) disappearance of either lucerne or wheat straw (*P* > 0.05) (Table 2).

**TABLE 1:** Fibrolytic enzyme specific activity in the sheep rumen contents pre- and postinoculation of R. albus and *R. flavefaciens*^*a*^

Enzyme	Enzyme sp act (mmol reducing sugars · mg protein^−1^ min^−1^)	
	Period 1, wk 27	Period 2, wk 33
CMCase	109 ± 46 A	278 ± 85 B
Xylanase	439 ± 127 A	1,140 ± 166 B

a Enzyme specific activity was measured from protein extracts prepared from the sheep rumen contents at the end of each period. Values are the means ± standard deviations (SD) of the data obtained for the two sheep and for rumen contents sampled pre- and postfeeding. Different letters after the values indicate significant difference at *P* < 0.01.

**TABLE 2:** Fiber degradation in the sheep rumen contents pre- and postinoculation of R. albus and *R. flavefaciens*^*a*^

Sheep and degradation process	Amt of lucerne (% (w/w) dry matter initial feed)		Amt of wheat straw cell walls (% (w/w) dry matter initial feed)	
	wk 23	wk 30	wk 25	wk 32
Sheep 421				
DMdis	20 ± 5 A	26 ± 2 B	19 ± 4 X	20 ± 2 X
NDFdis	12.5 ± 0.7 A	16.8 ± 0.2 B	18.1 ± 0.4	18.7 ± 0.2
ADFdis	9.2 ± 0.7 A	11.8 ± 0.2 B	10.9 ± 0.3	11.5 ± 0.4
Sheep 455				
DMdis	28 ± 1 A	26 ± 1 A	18 ± 2 X	7 ± 3 Y
NDFdis	17.5 ± 0.1 A	16.0 ± 0.2 B	19.2 ± 0.4 A	8.2 ± 1.1 B
ADFdis	13.7 ± 0.1 A	11.7 ± 0.1 B	11.2 ± 0.3 A	5.5 ± 0.2 B

a DMdis was measured from series of bags containing plant materials and incubated in the rumen for 24 h during either weeks 23 and 25 of period 1 or weeks 30 and 32 of period 2. The data obtained from the two animals were not averaged because at week 30 and week 32, the abundances of the three cellulolytic species had not evolved in the same manner in the two sheep as evidenced in Fig. 2. DMdis values are the means ± SD of the data obtained for each animal and each plant material. Different letters after the values indicate significant difference at *P* < 0.005. NDFdis and ADFdis values are the means of three fiber analysis determinations performed on pooled fermented residues retrieved from each bag series.

### Rumen metabolomics.

**FIG 7:**
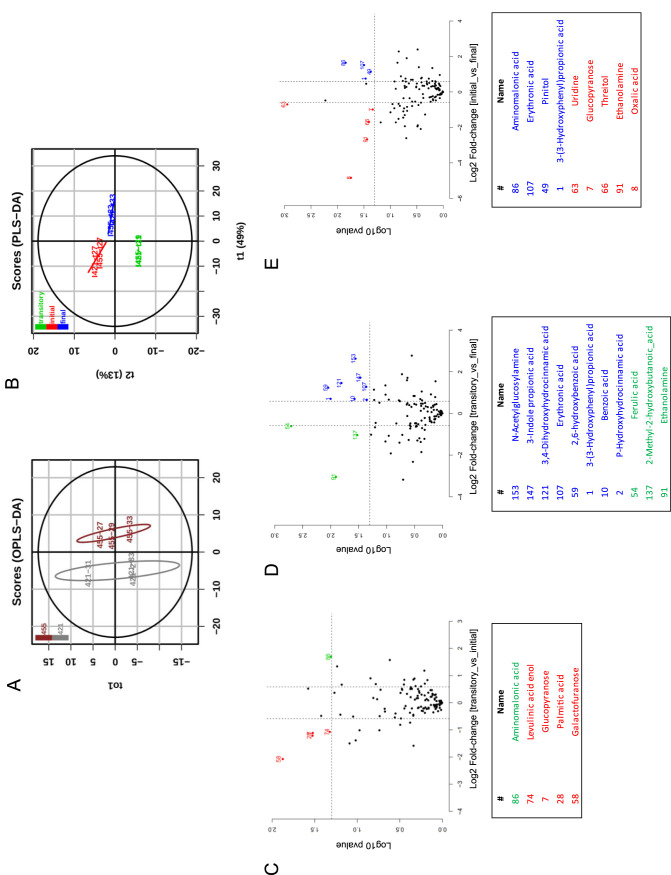
Metabolomic profiles according to animal (421 and 455) and sampling time. Sampling time was referred to as initial in red (week 27 for both animals), transitory in green (weeks 31 for 421 and week 29 for 455), and final in blue (week 33 for both animals). Variations in metabolomes were visualized by OPLS-DA model score plot for the animal response (A) and by PLS-DA score plot for the time response (B). Discriminant metabolites are presented in volcano plots showing pairwise comparisons between initial versus transitory (C), transitory versus final (D), and initial versus final (E) sampling times. The dashed lines mark the significance thresholds adjusted to a *P* value of <0.05 and to a log_2_ fold change of >0.6 or less than −0.6 corresponding to a fold change of >1.5 or less than −1.5. Significant metabolites obtained for each comparison were colored according to sampling time and were given a number for the corresponding metabolite presented in the table below each plot. In the tables, a positive or negative log_2_ fold change means a higher abundance or lower abundance, respectively, at the sampling time defined by its color. Only the metabolites (= variable) with a variable importance projection (VIP) of >1.0 in the PLS-DA analysis were considered relevant for time discrimination.

The rumen metabolome (Table S2) varied by animal and sample day (Fig. 7A and B). Profiles obtained before (week [wk] 27 [initial]) and immediately following the addition of the ruminococci (wk 30 [transitory]) revealed five discriminant metabolites that included a decrease in the free sugar’s glucopyranose and galactofuranose and the cellulose-derived keto acid levulinic acid (Fig. 7C). As the ruminococci outcompeted *F. succinogenes* (wk 30 versus wk 33 [final]), 11 discriminant metabolites were observed, including decreases in ferulic acid and increases in several phenolic metabolites (Fig. 7D). Comparisons of initial and final time points revealed nine discriminant metabolites (Fig. 7E), including an increase in 3-(3-hydroxyphenyl)propionic acid and a decrease in oxalic acid. Targeted metabolomic analyses of fermentation end products revealed that acetate and butyrate increased in their relative abundance following establishment of the ruminococci at the expense of propionate (Fig. S5).

10.1128/mBio.03533-20.3 TABLE S2 Raw metabolomic data obtained by GC-MS analysis of rumen fluid according to animal (421 and 455) and sampling time. Download Table S2, XLSX file, 0.03 MB. Copyright © 2021 Yeoman et al. 2021 Yeoman et al. https://creativecommons.org/licenses/by/4.0/ This content is distributed under the terms of the Creative Commons Attribution 4.0 International license.

10.1128/mBio.03533-20.8 FIG S5 Fluctuation of the SCFAs in rumen fluids of animal 421 (A and C, pre- and postfeeding, respectively) and of animal 455 (B and D, pre- and postfeeding, respectively). Download FIG S5, PDF file, 0.1 MB. Copyright © 2021 Yeoman et al. 2021 Yeoman et al. https://creativecommons.org/licenses/by/4.0/ This content is distributed under the terms of the Creative Commons Attribution 4.0 International license.

## DISCUSSION

The functional ecology of the major cellulolytic bacteria of the rumen was studied for the first time *in vivo* within a tractable animal model using complementary molecular, microbiological, enzymatic, and nutritional techniques. The animal model harbored in its rumen an immature microbiota that was limited to those microbes acquired during the first 17 h of contact with the mother (see Text S1 in the supplemental material). The lack of cultivable cellulolytic and methanogenic communities and measurable activities in our animal model allowed us to control the cellulolytic and methanogenic populations in a sequential manner by inoculating specific strains harboring these functions and then observe competition among three major rumen cellulolytic species. Multiple analyses revealed that R. albus became numerically, transcriptionally, and enzymatically dominant within 4 weeks of inoculation. This observation agrees with several *in vitro* coculture experiments that have also shown that R. albus was the most competitive in initial adhesion to, and growth, on cellulose (10, 12, 15). The high adherence of R. albus has been described *in vitro* to be the result of cell-surface-located type IV pili (16–18), CBM37-containing glycoside hydrolases (19, 20), and a glycocalyx (21). We observed high expression of type IV pilus genes by both R. albus and *R. flavefaciens* supporting an important role for these extracellular appendages in adhesion to cellulose *in vivo* (16–18, 22). Our data also support the importance of CBM37-harboring CAZymes by R. albus and cellulosome by *R. flavefaciens* for fiber breakdown *in vivo* with GH9 and GH48 family enzymes appearing to be preferred for ruminococcal cellulolytic function (7, 19, 20, 22).

10.1128/mBio.03533-20.1 TEXT S1 Molecular validation of the gnotobiotic sheep model (A) and population dynamics pre- and postfeed (B). Download Text S1, DOCX file, 0.04 MB. Copyright © 2021 Yeoman et al. 2021 Yeoman et al. https://creativecommons.org/licenses/by/4.0/ This content is distributed under the terms of the Creative Commons Attribution 4.0 International license.

R. albus has also been shown to produce *in vitro* a bacteriocin active against *R. flavefaciens* but not against *F. succinogenes* (23, 24). The gene encoding this bacteriocin in R. albus 8 has been described (25), and we found that it is very poorly transcribed in our animal model whatever the period (data not shown). Hence, bacteriocin production by R. albus cannot explain the fact that *R. flavefaciens* did not establish at high levels. *R. flavefaciens* was, however, able to numerically outcompete *F. succinogenes.* Despite the obvious ecological advantages of R. albus and the competitiveness of *R. flavefaciens* both *in vitro* and in our model, with the exception of two studies (26, 27), most studies performed with conventional sheep have instead found *F. succinogenes* to outnumber both *Ruminococcus* spp. (28–32). The difference between *in vitro* studies, our animal model, and these studies on conventional sheep may be attributable to differences in the broader ecology, where a newborn immature microbiota could be more ecologically favorable to the development of R. albus and *R. flavefaciens*, while a conventional adult microbiota may better favor *F. succinogenes* through unmeasured ecological interactions. This assumption agrees with studies of newborn calves and lambs in which cellulolytic ruminococci colonized the rumen earlier than *F. succinogenes* (33, 34). Diet may also influence the composition of both the cellulolytic and broader community (32, 35–37).

*F. succinogenes* expressed a more diverse CAZyme repertoire that included GH8, GH51, GH5, and GH9 family glycoside hydrolases. These genes are also highly expressed in *in vitro* continuous cultures of *F. succinogenes* S85 (38) and include a functionally characterized GH51 endoglucanase 2 (Eg2, CelF, and EgF) and the GH9 endoglucanase 1 (Cel2 and End1). Our results suggest that GH8 CAZymes may also be important to the fibrolytic function of *F. succinogenes* on lucerne. Additionally, four *F. succinogenes* genes (FSU_2078, FSU_2396, FSU_2397, and FSU_2502) have previously been proposed from *in vitro* proteomics studies to encode a four-protein complex within outer membrane vesicles (8). These vesicles are hypothesized to serve as vehicles of CAZymes that target plant biomass by means of the 180-kDa cellulose-binding protein encoded by 1 of 10 Fibro-slime-domain-containing gene, FSU_2502. Our metatranscriptomic data confirmed the high expression of these four genes *in vivo* and also suggest that OMV formation may involve additional genes, including a cluster of eight highly expressed genes (FSU_RS11370 = FSU_2396 to FSU_RS11405 = FSU_2404). As transcriptional changes do not necessarily reflect the translation and activity of the associated enzymes, we accompanied the metatranscriptomic analyses with functional analyses using *in sacco* incubations, zymograms, and metabolomics.

DM disappearance varied over time, corresponding to differences in the relative abundances of each cellulolytic species, but also between animals and by substrate and did not reflect measured CMCase and xylanase activities, which were 2.6-fold higher when ruminococci became dominant. This finding suggests that factors in addition to total fibrolytic enzyme activities must influence fibrolytic efficiency.

The resulting changes in fermentation end products, including a reduction in propionate and increases in acetate and butyrate, as the two *Ruminococcus* strains outcompeting *F. succinogenes* may impact the resulting productivity of the animal. Indeed, the amounts and profiles of short-chain fatty acids (SCFAs) formed in the rumen have consequences for the efficiency of energy utilization, production of methane, risks of ruminal acidosis, and composition of animal products (39, 40). *F. succinogenes* is an acetate and succinate producer, and succinate is a precursor of propionate (41). Our metatranscriptome data confirmed that two genes (FSU_RS14505 and FSU_RS14510) encoding fumarate reductase/succinate dehydrogenase involved in the succinate pathway were highly transcribed by *F. succinogenes in vivo*, which was not observed with the two ruminococci (data not shown). Hence, the disappearance of this species could in part explain the decrease in propionate. R. albus and *R. flavefaciens* produce acetate, formate, and hydrogen. All three products could account for the increase in acetate in rumen fluids knowing that formate and H_2_ can be utilized by acetogenic bacteria to produce acetate. We previously showed that acetogenic bacteria were active in similar animal models as long as methanogens did not colonize the ecosystem at high levels (14). The increase in butyrate may indicate unmeasured ecological interactions that favor growth of butyrate-producing bacteria as ruminococci become dominant.

In addition to SCFAs, several phenolic metabolites, including 3-(3-hydroxyphenyl)propionic acid were found to change in association with the abundance of the two ruminococci. These metabolites are catabolites of plant polyphenols, including hydroxycinnamic acids (42). It is possible that the ruminococci enhance the production of these molecules by hydrolyzing esterified hydroxycinnamic acids from lucerne hemicelluloses. While phenolic metabolites exhibit antimicrobial properties, R. albus and *R. flavefaciens* may be resistant to these effects, given findings that the dehydroxylated derivative of 3-(3-hydroxyphenyl)propionic acid, 3-phenylpropanoic acid has instead been shown to enhance their growth and fibrolysis *in vitro* (43–46).

In conclusion, this study describes for the first time *in vivo* competition between the three predominant cellulolytic rumen bacteria. It shows that global metabolic modifications in the rumen can be engendered by a simple disequilibrium in the cellulolytic community. It also provides *in vivo* evidence of the expression by the three cellulolytic bacterial species of different enzymatic and structural systems previously thoroughly studied *in vitro* that allow them to ensure their adhesion and hydrolytic function and survive in a very complex and competitive microbial digestive ecosystem.

## MATERIALS AND METHODS

### Ethics statement.

The experimental protocol was validated by the local ethics committee (Comité d’Ethique pour l’Experimentation Animale Auvergne) before beginning the trial, and the protocol was registered under the number CE18-08. Procedures were in accordance with the guidelines for animal research of the French Ministry of Agriculture and all other applicable national and European guidelines and regulations for experimentation with animals.

### Animal model.

The gnotobiotic sheep model has been described previously in detail (14, 47). Briefly, two INRAE line 401 lambs were born naturally and removed from their dams 17 h after birth. Lamb 421 (female) and lamb 455 (male) were then placed in a sterile isolator and reared under gnotobiotic conditions in the animal experimental facility of UMR MEDIS (Theix, Saint-Genes-Champanelle, France). Lambs were fed ultrahigh-temperature (UHT)-sterilized cow (Bos taurus) milk until they were 14 weeks old. At 2 weeks, they were also provided access to pelleted rations created from dehydrated lucerne (alfalfa) hay (7-mm diameter; SAFE, Augy, France). Lucerne pellets were sterilized by gamma irradiation (4 Mrad; Ionisos, Dagneux, France) prior to feeding. After 14 weeks, the animals were fed once daily at 8:00 a.m. with the lucerne pellets only. Sheep 421 and 455 were fitted within the sterile isolator with a permanent rumen plastisol cannula (diameter, 2.5 cm) at 17 and 18 weeks, respectively. Withdrawal of rumen contents and *in sacco* incubations began after the lambs had recovered from surgery at 22 weeks of age. The experiment ran for 12 weeks, and at the end of the experiment, sheep 421 and sheep 455 were both 34 weeks old and weighed 32 and 42 kg, respectively.

### Cellulolytic inoculation.

Lambs were monitored for the first 2 weeks after birth for the cultivable presence of cellulolytic and methanogenic activities, as well as for cultivable protozoa and fungi as previously described (14). None of these metabolisms or microbial groups were detected during this period. At 3 weeks of age, lambs were inoculated on 3 consecutive days with 20 ml (10^9^ cells ml^−1^) of a 2-day-old pure culture of Fibrobacter succinogenes S85 (ATCC 19169) fed on filter paper strips. The lambs were subsequently inoculated at 7 weeks of age with a pure culture of the ruminal methanogen *Methanobrevibacter* sp. 87.7 to enable hydrogenotrophy and ensure fibrolytic activity would not be inhibited by hydrogen buildup in this animal model (14, 47, 48). Finally, at 28 weeks of age, they were inoculated via cannula on 3 consecutive days with 160 ml (10^8^ cells ml^−1^) of 2-day-old pure cultures of both Ruminococcus flavefaciens FD1 (sourced from the Department of Animal Science, University of Illinois, Champaign-Urbana, IL, USA) and Ruminococcus albus 8 (sourced from USDA-National Center for Agricultural Utilization Research [NCAUR], Peoria, IL, USA) each supplied on filter paper strips. An overview of the animal trial and sampling procedure is presented in Fig. 1.

### Rumen sampling.

Rumen contents were withdrawn through the cannula on 2 consecutive days (each Tuesday and Wednesday) of weeks 22, 24, and 27 (*F. succinogenes* S85 was the only cellulolytic strain inoculated at these sample points) and on weeks 29, 31 and 33 (after R. albus 8 and *R. flavefaciens* FD1 had been inoculated) (Fig. 1). Sampling was performed before morning feeding (time zero [T0]) and 3 h after feeding (T3). T0 samples are reported with comparisons to T3 samples described in Text S1 in the supplemental material. The rumen content (50 ml) withdrawn on the first day of sampling was immediately used to determine ruminal pH and for culture-based microbial enumerations.

The rumen content (250 ml) withdrawn on the second day of sampling was treated as follows: a 50-ml aliquot was immediately mixed on ice with 100 ml RNAlater (Applied Biosystems, Courtaboeuf, France), aliquoted to four fractions of equal volumes, and stored at −80°C until nucleic acid extraction. A second 50-ml aliquot was kept on ice and treated as described below for polysaccharidase enzymatic analyses. A third 50-ml aliquot was frozen at −20°C (as sample saving) to determine the dry matter content of the rumen material. The remaining aliquot was centrifuged at 10,000 × *g* for 15 min at 4°C, and 1 ml of supernatant was taken and stored at −80°C for metabolomic analyses.

### Enumeration of microbial communities.

Total cultivable bacteria, total cultivable cellulolytic bacteria, and total cultivable methanogens were enumerated by using the most probable number (MPN) method as described previously (14).

### *In sacco* digestibility assays.

To evaluate plant substrate degradation in our model, we used the lucerne (alfalfa) pellets, as provided in the animal diet, and cell walls prepared as described previously (49) from ground and sieved (160 to 400 μm) wheat straw. Cell wall residues were prepared using an Soxhlet extractor by refluxing in 1:2 ethanol-toluene and then 95°C ethanol until the extracts became colorless (49). Nylon bags (R510 *in situ* bags [100 × 50 mm; porosity of 50 μm]; Ankom Technology, NY, USA) were filled with either 1 g dry matter (DM) of the lucerne pellets or 0.5 g DM of wheat straw cell walls. *In sacco* incubations of lucerne bags were carried out for both sheep beginning on weeks 23 (post-fibrobacter inoculation but pre-ruminococci inoculation) and 30 (post-ruminococci inoculation), while incubations of wheat cell wall bags began on weeks 25 (also post-fibrobacter inoculation but pre-ruminococci inoculation) and 32 (post-ruminococci inoculation; Fig. 1). During 8 consecutive days, two bags were introduced into the rumen before morning feeding and removed after 24 h of incubation. One bag was then immediately washed in RNase-free phosphate-buffered saline (PBS) until the buffer was clear. It was then placed in a sterile tube containing a mixture of 20 ml RNAlater (Applied Biosystems, Courtaboeuf, France) and 10 ml RNase-free water and frozen at −80°C for subsequent molecular analyses. The other bag was washed with tap water until the water was visibly clear and then frozen at −20°C for subsequent fiber composition analysis. Extra bags were also incubated to provide materials for electron microscopy observations of adhering bacteria, as described below.

Dry matter disappearance was determined by measuring changes in the weight of nylon bags between insertion and after removal and drying at 60°C for at least 72 h. Contents of the bags corresponding to the same plant substrate, same week of incubation, and same animal were pooled together for analyses. Residues were analyzed for neutral detergent fiber (NDF) and acid detergent fiber (ADF) contents using the Ankom fiber analyzer (Ankom Technology, NY, USA). Unincubated (control) bags were treated identically and used to calculate the base lines of DM, NDF, and ADF disappearances (DMdis, NDFdis, and ADFdis, respectively).

### Enzyme activity assays and zymograms.

Microbial enzymes from rumen contents were recovered as follows: a freshly collected rumen sample (50 ml) was centrifuged at 10,000 × *g* for 15 min at 4°C, and the pellet was washed several times with an anaerobic mineral solution (50) until supernatant was clear. The washed pellet containing rumen microbes and lucerne residues was then frozen at −80°C until analysis. Thawed pellets (1 g) from weeks 27 and 33 (end of each period) were mixed with 5 ml phosphate buffer (50 mM, pH 7) containing the Complete protease inhibitor cocktail (Roche, Boulogne-Billancourt, France). Cell lysis was performed by sonication (four pulses of 30 s at 20 KHz and 60 W, separated by 1-min pause on ice). The resulting mixture was then centrifuged at 8,000 × *g* for 30 min at 4°C, and the supernatant was assayed for polysaccharidase specific activity and zymogram analysis as previously described (21), using carboxymethyl cellulose (medium viscosity) and oatspelt xylan (Sigma-Aldrich, Saint-Quentin-Fallavier, France) as the substrates.

### Electron microcopy.

The lucerne and wheat straw cell walls from the *in sacco* incubations were fixed with 3% glutaraldehyde in PBS for 1h at 4°C. They were then treated and examined with a Philips SEM 505 scanning electron microscope as described previously (21).

### Nucleic acid extraction.

Rumen contents and *sacco* residues, both in suspension in RNAlater (Applied Biosystems, Courtaboeuf, France) were thawed on ice and centrifuged at 15,000 × *g* for 15 min at 4°C, and the resulting supernatant was discarded. Pellets from *sacco* residues corresponding to the same plant substrate, same week of incubation, and same animal were pooled to obtain sufficient quantities of material for nucleic acid extraction. DNA was extracted in triplicate from 0.25 g of pellet using an MP Biomedical DNA extraction and purification kit (Fast DNA Spin kit and Gene Clean Turbo; MP Biomedicals, Illkirch, France) following the manufacturer’s recommendations. RNA was extracted in quadruplicate from 0.70 g of rumen content pellet using a Nucleospin RNAII kit (Macherey-Nagel, Hoerdt, France) following the supplier’s instructions with the following exceptions. (i) During the cell lysis step, approximately 160-mg zirconia beads of 0.1-mm diameter (Bio Spec Products, Bartlesville, OK, USA) were added to the sample in addition to RA1 buffer and β-mercaptoethanol, for bead-beating (twice for 30 s each time at maximum power of 6.5) using the FastPrep instrument (MP Biomedicals, Illkirch, France). (ii) During the washing step, buffer RA2 was replaced by buffer FW1 as recommended by the supplier. Eluted RNA was submitted to an additional RNase-free DNase treatment (provided in the RNAII kit). RNA integrity was assessed using an Agilent 2100 bioanalyzer as previously described (51), and all samples were found to have a RNA integrity number (RIN) of >8. Nucleic acid concentrations were estimated by absorbance at 260 nm (NanoDrop ND-1000). They were kept at −80°C until utilization.

### Quantitative real-time PCR.

Quantitative PCR (qPCR) targeting total bacteria, methanogenic archaea, and each of the cellulolytic species—R. albus, *R. flavefaciens*, and *F. succinogenes*—was performed using SYBR green chemistry with a Mastercycler ep realplex 2S (Eppendorf, Le Pecq, France) in 96-well plates (Twin.tec plate 96 skirted; Eppendorf). Results were analyzed using realplex version 2.0 software (Eppendorf). The qPCR conditions (i.e., primers, reaction mixture, program, standard curves) have been described previously (29), except that we used the IQ SYBR Green Supermix (Bio-Rad, Marnes-la-Coquette, France). qPCRs were performed in triplicate from three separate extracts of DNA per sample (biological replicates). Results were expressed as *rrs* copies of microbial target per gram (dry matter) of sample (centrifuged rumen content or *sacco* residue).

### Genomic reference data.

Representative genomes from each of the cellulolytic species have previously been reported for *R. flavefaciens* 17 (52), R. albus 8 (53), and Fibrobacter succinogenes S85 (54). *Methanobrevibacter* sp. 87.7 was sequenced at Montana State University via a single run of an Illumina MiSeq. DNA was extracted using the DNeasy PowerLyzer microbial kit (Qiagen, Germantown, MD, USA), and genomic data were assembled using SPAdes v. 3.6.2 and gave 500-fold coverage. Genomic data were annotated using NCBI’s Prokaryotic Genome Annotation Pipeline (https://www.ncbi.nlm.nih.gov/genome/annotation_prok/) and are deposited in GenBank under NZ_MRCT01000000 and BioProject PRJNA339853. The four genomes used as reference in this study are presented in Table 3.

**TABLE 3:** Genomes used in this study

Strain	BioProject ID	NCBI accession no.
Fibrobacter succinogenes subsp. *succinogenes* S85	PRJNA224116	NC_017448.1
*Methanobrevibacter* sp. 87.7	PRJNA339853	MRCT00000000.1
Ruminococcus albus 8	PRJNA224116	NZ_ADKM00000000.2
Ruminococcus flavefaciens FD-1	PRJNA224116	NZ_ACOK01000031.1

### Metagenomic and metatranscriptomic analyses.

Rumen samples withdrawn before feeding (time zero [T0]) at weeks 27, 31, and 33 for animal 421 and weeks 27, 29, and 33 for animal 455 (Fig. 1) were subjected to metagenomic and metatranscriptomic analyses. Triplicate DNA and quadruple RNA extracts were each pooled by animal and time point. RNA extracts were transcribed to cDNA by first strand cDNA synthesis, and both DNA and cDNA were independently sequenced with one lane each of paired-end 2 × 125 nucleotide (nt) HiSeq 2500 (Illumina) sequencing.

Sequence data were quality and adapter trimmed using Trimmomatic v 0.33 (55). Metatranscriptome data were then further preprocessed using SortMeRNA v 2.1b (56) to separate sequences into rRNA and non-rRNA fractions. Full metagenomic data and the rRNA and non-rRNA subset of metatranscriptomic data from the SortMeRNA analysis were aligned to all four reference genomes as a single index file, using the Burrows-Wheeler Aligner v 0.7.15 (57). Coverage data for all alignments (used for downstream visualization) were generated using deepTools v. 2.5.0.1 (58) and visualized using the Integrative Genomics Viewer v2.3.92 (59). Basic expression normalization to reads per kilobase per million (RPKM) was performed using the edgeR v3.18.1 package in R v3.4.0 (60). Heat-maps were generated using the R package, ggplots on expression data scaled to the standard deviation from the mean. *De novo* CAZy-specific functional analysis was performed using HMMER v 3.1b2 (61) and the Release 5.0 protein motifs obtained from dbCAN (62). Read counts for all coding sequence annotation from each genome were generated using featureCounts v1.9.2 (63). All metagenomic and metatranscriptomic data were aligned using DIAMOND v. 0.8.37 (64) against the NCBI nonredundant protein database (July 2017). Taxonomic and functional information assignment as well as initial analyses and summary figures for taxonomic and functional classification were generated using MEGAN v6.8.13 (65). Metagenomic and metatranscriptomic data are deposited in GenBank under BioProject PRJNA646650.

### Metabolomic analyses.

Using complementary samples to those utilized for metagenomic and metatranscriptomic analyses, 1 ml of the rumen supernatant (collected as described above) was dried and derivatized as previously described (66). A 5-μl aliquot of an internal standard (C_31_ fatty acid) was added to each sample prior to derivatization. Samples were injected with a split ratio of 7:1 into a gas chromatography (GC)-mass spectrometry (MS) system, consisting of an Agilent 7890A (Agilent Inc., Palo Alto, CA, USA) gas chromatograph, an Agilent 5975C mass selective detector, and an Agilent 7683B autosampler. Gas chromatography was performed on 60-m HP-5MS columns with 0.25-mm inner diameter and 0.25-mm film thickness (Agilent Inc., Palo Alto, CA, USA) and with an injection temperature of 2,500°C, the interface set at 2,500°C, and the ion source adjusted to 2,300°C. The helium carrier gas was set at a constant flow rate of 1.5 ml min^−1^. The temperature program of 5-min isothermal heating at 700°C, followed by an oven temperature increase of 50°C min^−1^ to 3,100°C and a final 20 min at 3,100°C. The mass spectrometer was operated in positive electron impact mode (EI) at 69.9 eV ionization energy in the *m/z* 30 to 800 scan range. The spectra of all chromatogram peaks were then compared with electron impact mass spectrum libraries NIST08 (National Institute of Standards and Technology [NIST], MD, USA) and WILEY08 (Palisade Corporation, NY, USA) and a custom library of the University of Illinois Roy J. Carver Metabolomics Center. To allow direct comparisons between samples, all data were normalized to the internal standard in each chromatogram. The chromatograms and mass spectra were evaluated using the MSD ChemStation (Agilent, Palo Alto, CA, USA) and AMDIS (NIST, Gaithersburg, MD, USA). The retention time and mass spectra were implemented within the AMDIS method formats.

Analysis of volatile fatty acid (VFA) was performed without derivatization on rumen fluids using gas chromatography as previously described (67). Fermentation end products were assessed by comparing prefeeding (0-h) and 3-h postfeeding profiles throughout the animal experiment.

### Statistical analyses.

A Student’s *t* test was used to compare DM disappearance, as well as polysaccharidase specific activities in animals between period 1 (weeks 22 to 27) with *F. succinogenes* only and period 2 (weeks 28 to 33) after inoculation of cellulolytic ruminococci. Differences were considered significant at *P* < 0.05.

Metabolomic data were analyzed by using a partial least-squares discriminant analysis (PLS-DA) model to visualize variations of metabolites among experimental time points (initial [week 27], transitory [weeks 29 to 31], and final [week 33]) and an orthogonal PLS-DA for the two animals (421 and 455). The raw matrix with metabolite concentrations was log_10_ transformed before analyzing the data with the ropls R package (68, 69). To identify the metabolites responsible for discrimination between two time points (initial versus transitory, transitory versus final, and initial versus final), a pairwise comparative analysis using a *t* test was used to test the statistical significance. Only the variables (metabolites) with a variable importance projection (VIP) of >1.0 in the PLS-DA analysis were considered relevant for time discrimination. These tests were performed using the MetaboDiff R package (70) and were normalized using the variance stabilization method (71) before the differential analyses.

### Data availability.

The data sets supporting the results of this article are included within the article and its additional supplemental material. The genome sequence of *Methanobrevibacter* sp. 87.7 was deposited in GenBank under NZ_MRCT01000000 and BioProject PRJNA339853. The metagenome and metatranscriptome data are available in GEO data sets at NCBI (http://www.ncbi.nlm.nih.gov/) under BioProject PRJNA646650.

10.1128/mBio.03533-20.9 FIG S6 Overall taxonomic distribution from metagenomic data. Metagenomic reads were aligned to the July 2017 NCBI nonredundant database using DIAMOND 0.8.37, and taxonomic and functional assignments were derived from the resulting protein hits using MEGAN v6.8.13. Counts were then normalized within MEGAN during comparative analysis and transformed to log scale prior to plotting using MEGAN. Plots represent reads were agglomerated to the taxonomic family level (NCBI classification). Download FIG S6, PDF file, 0.2 MB. Copyright © 2021 Yeoman et al. 2021 Yeoman et al. https://creativecommons.org/licenses/by/4.0/ This content is distributed under the terms of the Creative Commons Attribution 4.0 International license.

10.1128/mBio.03533-20.10 FIG S7 Overall taxonomic distribution from metatranscriptomic data. Transcriptomic reads were aligned to the July 2017 NCBI nonredundant database using DIAMOND 0.8.37, and taxonomic and functional assignments were derived from the resulting protein hits using MEGAN v6.8.13. Counts were then normalized within MEGAN during comparative analysis and transformed to log scale prior to plotting using MEGAN. Plots represent reads were agglomerated to the taxonomic family level (NCBI classification). Download FIG S7, PDF file, 0.2 MB. Copyright © 2021 Yeoman et al. 2021 Yeoman et al. https://creativecommons.org/licenses/by/4.0/ This content is distributed under the terms of the Creative Commons Attribution 4.0 International license.
