# Supplementary material for: In Vivo Competitions between Fibrobacter succinogenes, Ruminococcus flavefaciens, and Ruminoccus albus in a Gnotobiotic Sheep Model Revealed by Multi-Omic Analyses
Source: mBio. 2021 Mar 3;12(2):e03533-20. doi: 10.1128/mBio.03533-20 (PMC8092306; doi:10.1128/mBio.03533-20)
Supplement: FIG S1 [file mBio.03533-20-sf001.pdf]

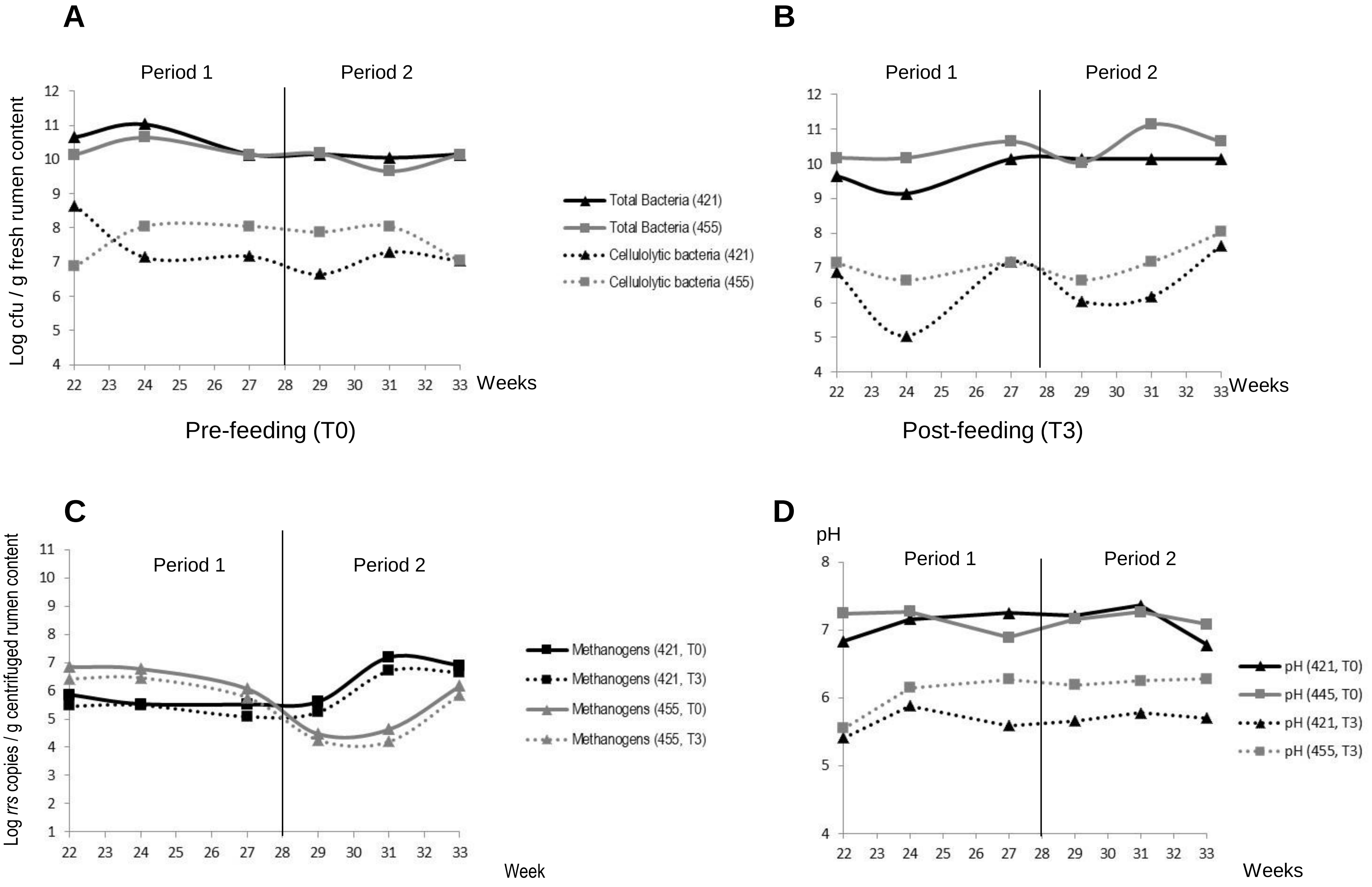

Figure S1: Dynamics of cultivable bacteria before morning feeding (A) and 3h after feeding (B), of methanogens evaluated by quantitative PCR (C) and pH monitoring (D).
