# Supplementary material for: In Vivo Competitions between Fibrobacter succinogenes, Ruminococcus flavefaciens, and Ruminoccus albus in a Gnotobiotic Sheep Model Revealed by Multi-Omic Analyses
Source: mBio. 2021 Mar 3;12(2):e03533-20. doi: 10.1128/mBio.03533-20 (PMC8092306; doi:10.1128/mBio.03533-20)
Supplement: FIG S3 [file mBio.03533-20-sf003.pdf]

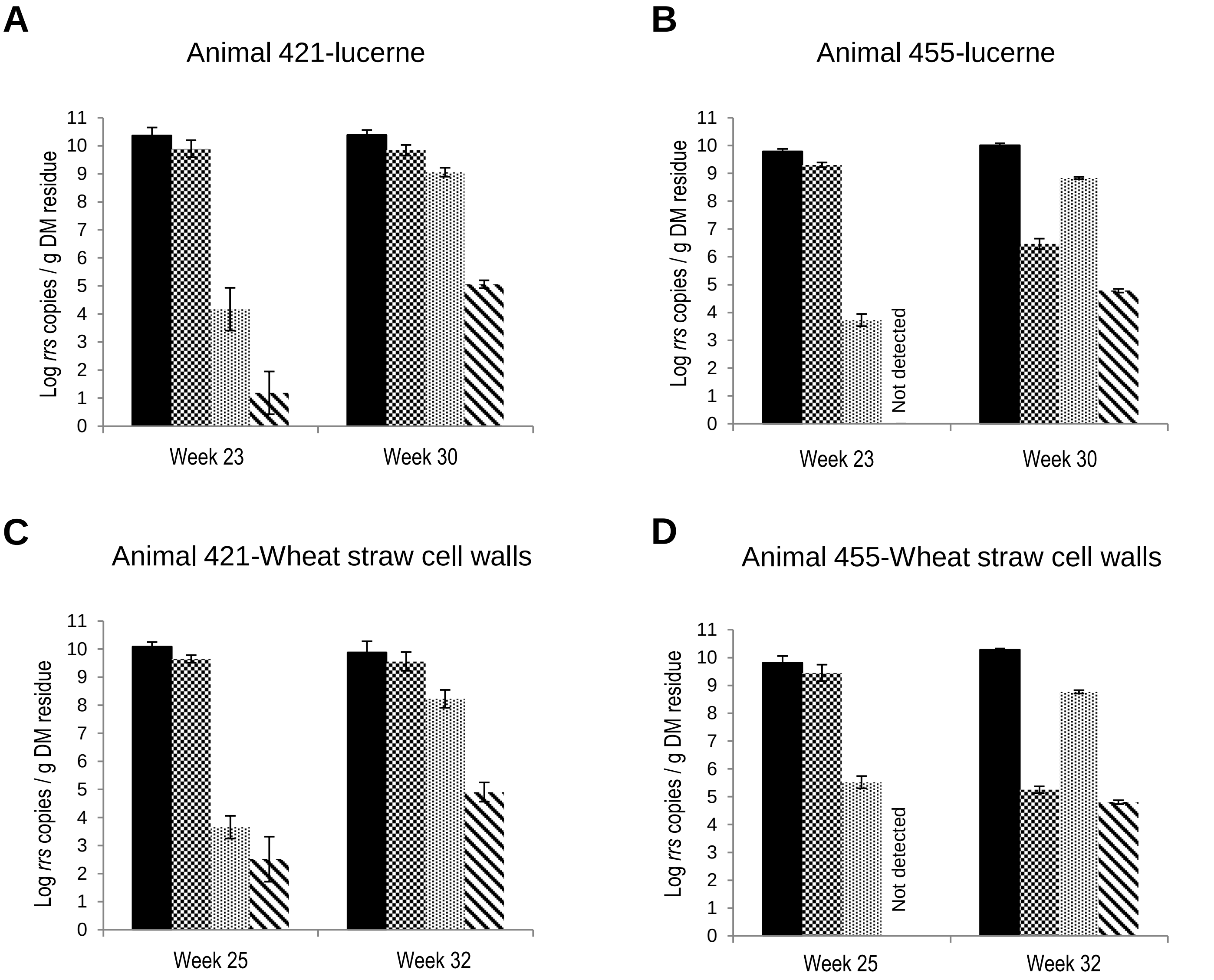

Figure S3: Quantitative determination of adhering microbes to two different plant materials
