# Supplementary figures and images for: In Vivo Competitions between Fibrobacter succinogenes, Ruminococcus flavefaciens, and Ruminoccus albus in a Gnotobiotic Sheep Model Revealed by Multi-Omic Analyses
Source: mBio. 2021 Mar 3;12(2):e03533-20. doi: 10.1128/mBio.03533-20 (PMC8092306; doi:10.1128/mBio.03533-20)

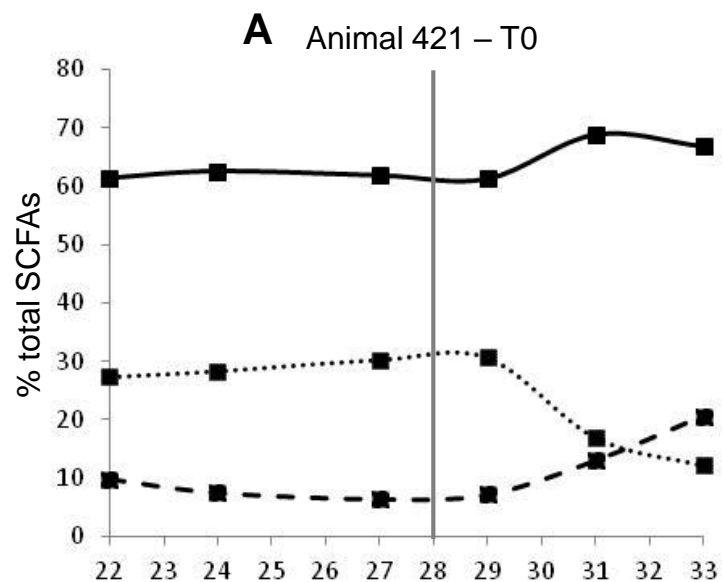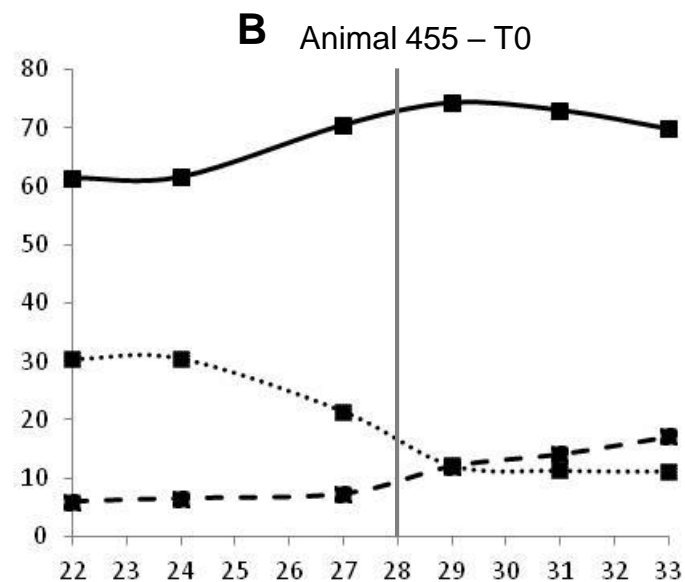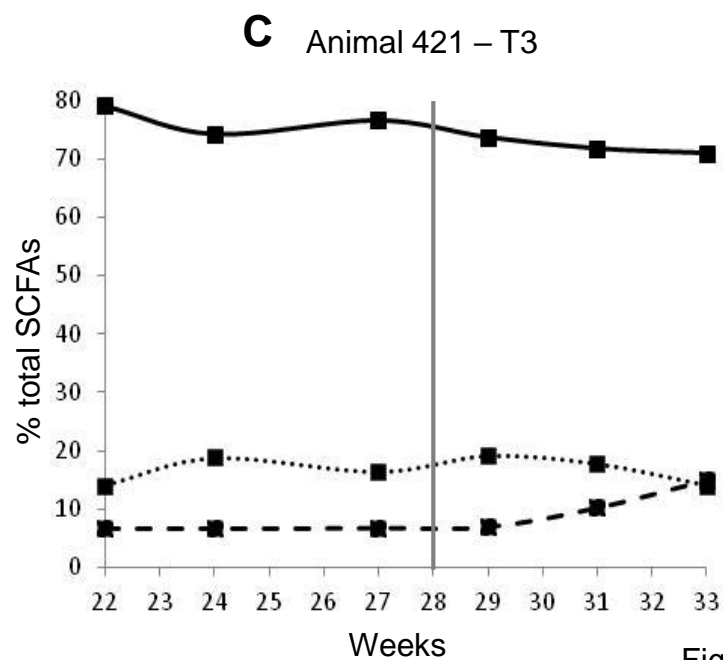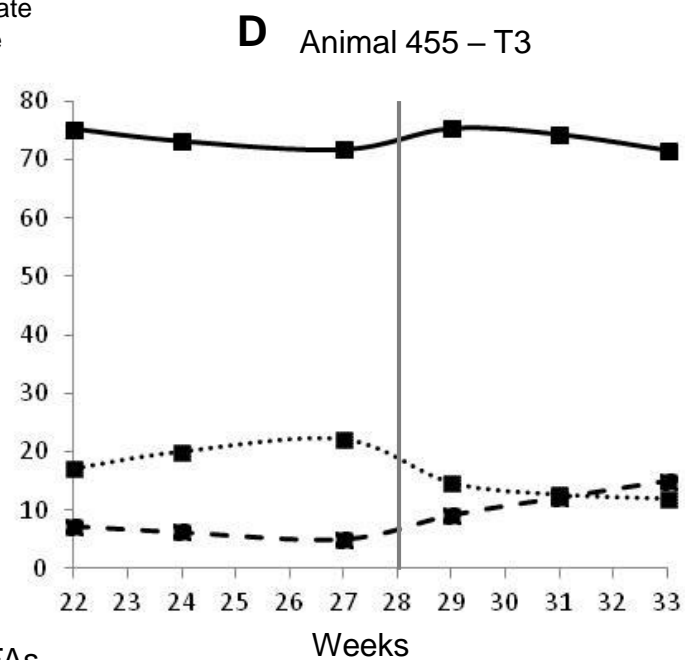

Figure S5: SCFAs

Supplement: FIG S5 [file mBio.03533-20-sf005.pdf]

Figure S6: Overall taxonomic distribution from metagenomic data

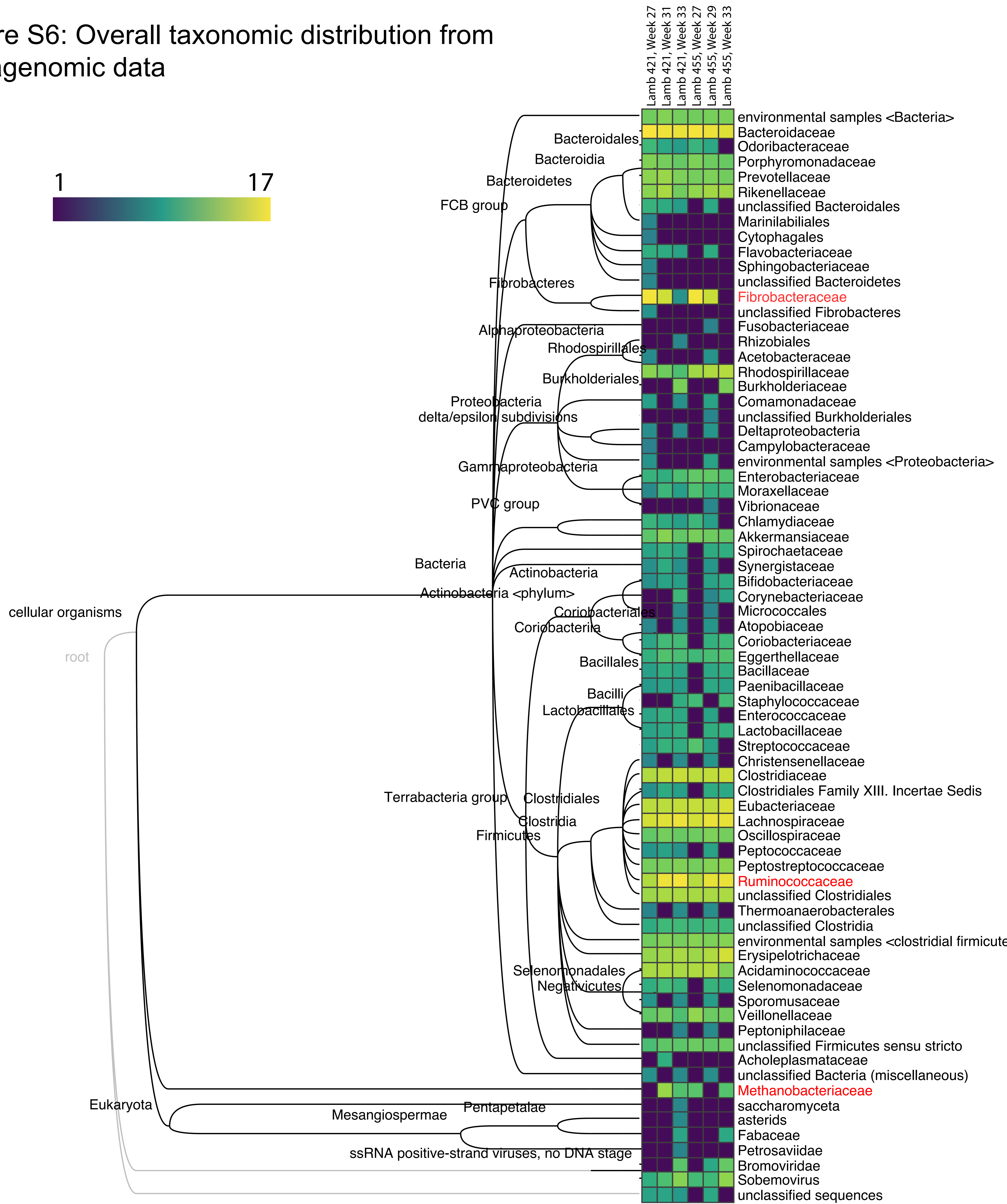

Supplement: FIG S6 [file mBio.03533-20-sf006.pdf]

Figure S7: Overall taxonomic distribution from metatranscriptomic data

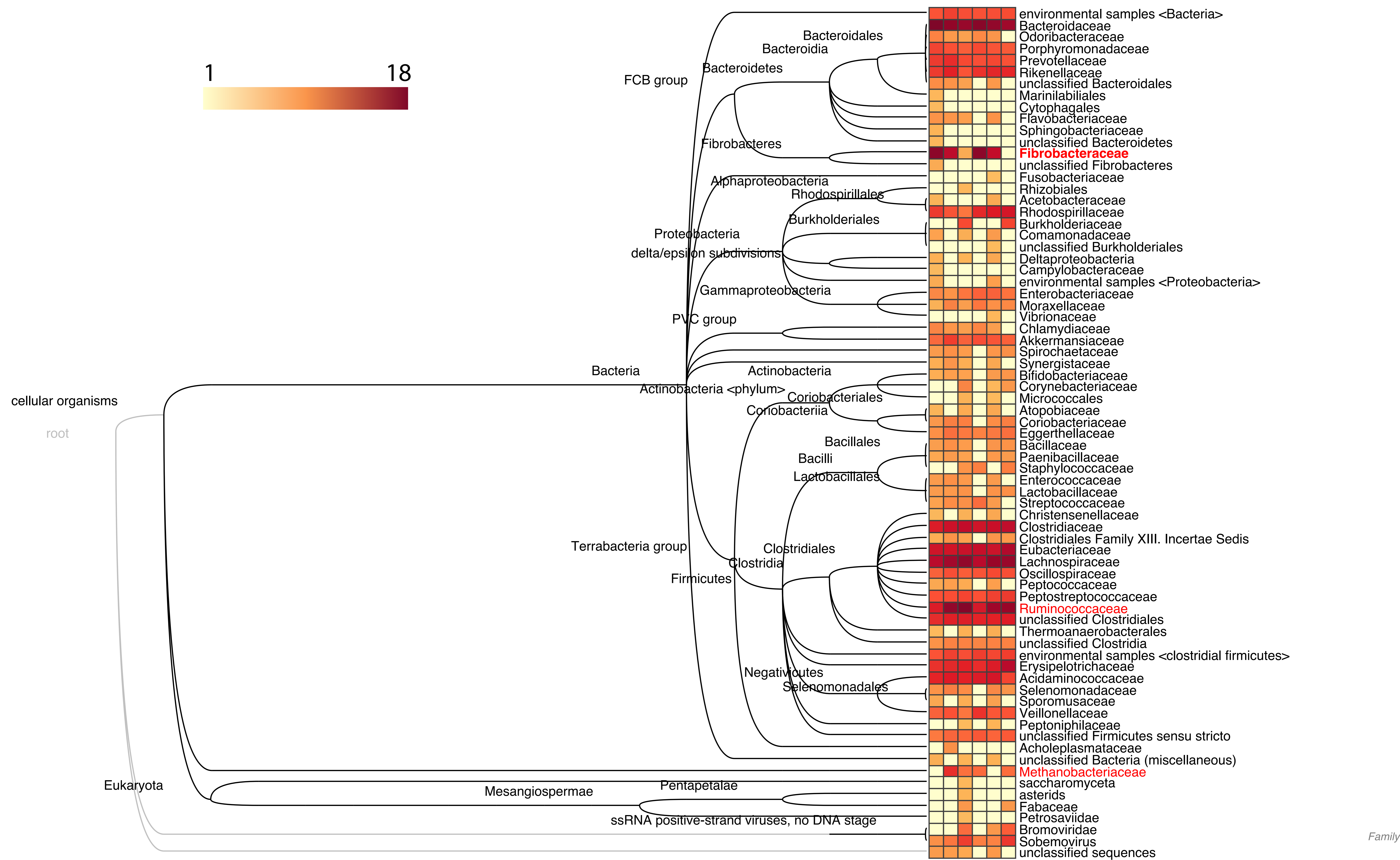

Supplement: FIG S7 [file mBio.03533-20-sf007.pdf]
