# Supplementary material for: In Vivo Competitions between Fibrobacter succinogenes, Ruminococcus flavefaciens, and Ruminoccus albus in a Gnotobiotic Sheep Model Revealed by Multi-Omic Analyses
Source: mBio. 2021 Mar 3;12(2):e03533-20. doi: 10.1128/mBio.03533-20 (PMC8092306; doi:10.1128/mBio.03533-20)
Supplement: TEXT S1 [file mBio.03533-20-s0001.docx]

**Supplementary text 1:**

1. **Molecular validation of the gnotobiotic sheep model**

Gnotobiotic sheep models are unique and methodologically difficult to produce, as is evidenced by research having only successfully been performed and published from a single laboratory worldwide to date. In this study, our aim was not to produce axenic animals born by C-section and further inoculated with a limited number of known microorganisms but to obtain animal models with an immature but complex microbiota (<24h-old) devoid of eukaryotes, archaea, and cellulolytic bacteria. As for axenic animals, this gnotobiotic sheep model requires to be raised under axenic conditions. While we set out to perform this study in six animals only two lambs were successfully derived and maintained under sterile conditions over the 8 months from birth to the conclusion of the experiment. In addition, contrary to rodent gnotobiotic models where axenic animals can be used as negative controls, axenic sheep cannot be used as negative control if fed a solid fiber-based diet because the symbiosis between the rumen microbiota and the host is essential for animal viability. Indeed, ruminant growth and maintenance depend on the rumen microbiota to retrieve energy (short chain fatty acids) from the complex structural fibers present in its solid food diet, in addition to microbial proteins (1).

As expected, metagenome and metatranscriptome analyses identified bacterial taxa in addition to the inoculated species that appeared transcriptionally active in this animal model (Figs. S6, S7). Recent studies have demonstrated rumen microbial colonization within the first 24 hours after birth (2-5), including from maternal sources, therefore it is likely these microbes were acquired in the 17h prior to the removal of newborn lambs from their dams. According to our metagenome (Fig. S6) and metatranscriptome (Fig. S7) analyses, the microbiota composition observed agreed with data obtained on newborn calves determined by 16S metabarcoding (2, 5). Although previous as well as the present work detected ruminal *R. albus* and *R. flavefaciens* 16S rDNA sequences in the first 24h of life using qPCR (2, 6), the microbiota of our sheep model raised in sterile isolators was found to be devoid of cultivable cellulolytic organisms and the corresponding biological activities, consistent with similar previous observations (7-10). Likewise, while methanogens have been detected by qPCR and sequencing of 16S rRNA genes in 1-day-old ruminants previously (6, 11), and were observed in metagenomic data, we were unable to cultivate methanogenic Archaea from our lambs prior to the introduction of *M. sp.* 87.7. An absence of methanogenic activity has also previously been described in 24 h old lambs (9, 10).

Metagenomic and metatranscriptomic analyses identified several bacterial families with high levels of mRNA expression throughout the study period. These families were *Bacteroidaceae, Porphyromonaceae, Prevotellaceae* and *Rikenellaceae* (Bacteroidetes phylum), *Rhodospirillaceae, Enterobacteriaceae* (Proteobacteria phylum), *Akkermansiaceae* (*Verrucomicrobia* phylum), *Clostridiaceae, Eubacteriaceae, Oscillospiraceae, Peptostreptococcaceae Erysipelotrichaceae, Acidaminococcaceae* (Firmicutes Phylum). We did not detect anaerobic eukaryotic organisms *i.e.* protozoa and fungi, which is consistent with previous studies of their establishment in the rumen (12, 13).

Interestingly, within the *Bacteroidetes* phylum, *Bacteroidaceae* were very abundant and more transcriptionally active than *Prevotellaceae* throughout the animal experiment despite *Prevotella* having been shown in several studies to be the dominant genera in adult ruminants (14-17). A shift from *Bacteroidaceae* to *Prevotellaceae* as an animal matures has previously been observed by Mizrahi and Jami (18) and hypothesized to be due to the introduction of plant-based feed. Our findings suggest additional ecological factors may contribute to the fitness of *Prevotellaceae* in adult animals.

Furthermore, *Burkholderiaceae* and *Lachnospiraceae* families were more active at the transcriptomic level when the rumen was dominated by the cellulolytic ruminococci. In fact, the *Lachnospiraceae* family increased in parallel with the two ruminococcal strains. Members of the *Lachnospiraceae* family, including *Butyrivibrio sp, Pseudobutyrivibrio sp. Roseburia sp. Coprococcus sp. Anaerostipes sp.* are abundant in the conventional rumen and are of interest as the major butyrate-producers (19).

1. **Population dynamics pre- and post-feed**

The 16S rDNA copies of total bacteria/ g of rumen content (RC) decreased slightly between pre-feed samples (T0; 10^10.4 ± 0.12^) and those taken 3h post-feeding (T3; 10^10.2 ± 0.16^) (*P* = 0.01) (Fig. 2), however, MPN results did not differ significantly (Fig. S1A, S1B). Cultivable cellulolytic numbers also decreased between T0 (10^7.5 ± 0.6^ cells / ml) and T3 (10^6.8 ± 0.8^ cells / ml) (*P* = 0.015; Fig. S1A, S1B). The reduction in both total cultivable and cultivable cellulolytic bacteria between T0 and T3 corresponded to increases in total rumen short chain fatty acid (SCFA; *P* =4.9x10^-9^) from 53 ± 17 mM (T0) to 127 ± 23 mM (T3), and a reduction in ruminal pH (*P* = 4.6x10^-11^) from pH 7.12 ± 0.19 to pH 5.89 ± 0.32 (Fig. S1D).

*F.* *succinogenes* was always found to be numerically lower in T3 samples than those taken at T0, however, this was only found to be significant prior to the inoculation of the ruminococci (*P* =0.027) when *F.* *succinogenes* numbers were stable. Following inoculation, *R. albus* was also always numerically lower at T3 than T0, while *R. flavefaciens* 16S rDNA copies were higher at T3 relative to those at T0, again neither of these observations were found to be significant, likely due to the gradual increase in numbers of each between sample points (Fig. 2).

1. Loor JJ, Elolimy AA, McCann JC. 2016. Dietary impacts on rumen microbiota in beef and dairy production. Anim Front 6:22-29.

2. Jami E, Israel A, Kotser A, Mizrahi I. 2013. Exploring the bovine rumen bacterial community from birth to adulthood. ISME J 7:1069-79.

3. Jiao J, Huang J, Zhou C, Tan Z. 2015. Taxonomic identification of ruminal epithelial bacterial diversity during rumen development in goats. Appl Environ Microbiol 81:3502-9.

4. Jiao J, Li X, Beauchemin KA, Tan Z, Tang S, Zhou C. 2015. Rumen development process in goats as affected by supplemental feeding v. grazing: age-related anatomic development, functional achievement and microbial colonisation. Br J Nutr 113:888-900.

5. Yeoman CJ, Ishaq SL, Bichi E, Olivo SK, Lowe J, Aldridge BM. 2018. Biogeographical differences in the influence of maternal microbial sources on the early successional development of the bovine neonatal gastrointestinal tract. Sci Rep 8:3197.

6. Guzman CE, Bereza-Malcolm LT, De Groef B, Franks AE. 2015. Presence of selected methanogens, fibrolytic bacteria, and proteobacteria in the gastrointestinal tract of neonatal dairy calves from birth to 72 Hours. PLoS One 10:e0133048.

7. Fonty G, Gouet P, Nebout JM. 1989. Development of the cellulolytic microflora in the rumen of lambs transferred into sterile isolators a few days after birth. Can J Microbiol 35:416-22.

8. Fonty G, Jouany JP, Chavarot M, Bonnemoy F, Gouet P. 1991. Development of the rumen digestive functions in lambs placed in a sterile isolator a few days after birth. Reprod Nutr Dev 31:521-8.

9. Chaucheyras-Durand F, Masseglia S, Fonty G, Forano E. 2010. Influence of the composition of the cellulolytic flora on the development of hydrogenotrophic microorganisms, hydrogen utilization, and methane production in the rumens of gnotobiotically reared lambs. Appl Environ Microbiol 76:7931-7937.

10. Gagen EJ, Mosoni P, Denman SE, Al Jassim R, McSweeney CS, Forano E. 2012. Methanogen colonisation does not significantly alter acetogen diversity in lambs Isolated 17 h after birth and raised aseptically. Microb Ecol 64:628-640.

11. Wang Z, Elekwachi CO, Jiao J, Wang M, Tang S, Zhou C, Tan Z, Forster RJ. 2017. Investigation and manipulation of metabolically active methanogen community composition during rumen development in black goats. Sci Rep 7:422.

12. Fonty G, Gouet P, Jouany JP, Senaud J. 1983. Ecological factors determining establishment of cellulolytic bacteria and protozoa in the rumens of meroxenic lambs. J Gen Microbiol 129:213-23.

13. Fonty G, Gouet P, Jouany JP, Senaud J. 1987. Establishment of the microflora and anaerobic fungi in the rumen of lambs. J Gen Microbiol 133:1835-1843.

14. Stevenson DM, Weimer PJ. 2007. Dominance of *Prevotella* and low abundance of classical ruminal bacterial species in the bovine rumen revealed by relative quantification real-time PCR. Appl Microbiol Biotechnol.

15. Bekele AZ, Koike S, Kobayashi Y. 2010. Genetic diversity and diet specificity of ruminal *Prevotella* revealed by 16S rRNA gene-based analysis. FEMS Microbiol Lett 305:49-57.

16. Comtet-Marre S, Parisot N, Lepercq P, Chaucheyras-Durand F, Mosoni P, Peyretaillade E, Bayat AR, Shingfield KJ, Peyret P, Forano E. 2017. Metatranscriptomics reveals the active bacterial and eukaryotic fibrolytic communities in the rumen of dairy cow fed a mixed diet. Front Microbiol 8:67.

17. Chaucheyras-Durand F, Ameilbonne A, Auffret P, Bernard M, Mialon MM, Duniere L, Forano E. 2019. Supplementation of live yeast based feed additive in early life promotes rumen microbial colonization and fibrolytic potential in lambs. Sci Rep 9:19216.

18. Mizrahi I, Jami E. 2018. Review: The compositional variation of the rumen microbiome and its effect on host performance and methane emission. Animal 12:S220-S232.

19. Seshadri R, Leahy SC, Attwood GT, Teh KH, Lambie SC, Cookson AL, Eloe-Fadrosh EA, Pavlopoulos GA, Hadjithomas M, Varghese NJ, Paez-Espino D, Perry R, Henderson G, Creevey CJ, Terrapon N, Lapebie P, Drula E, Lombard V, Rubin E, Kyrpides NC, Henrissat B, Woyke T, Ivanova NN, Kelly WJ, Palevich N, Janssen PH, Ronimus RS, Noel S, Soni P, Reilly K, Atherly T, Ziemer C, Wright AD, Ishaq S, Cotta M, Thompson S, Crosley K, McKain N, Wallace RJ, Flint HJ, Martin JC, Forster RJ, Gruninger RJ, McAllister T, Gilbert R, Ouwerkerk D, Klieve A, Al Jassim R, Denman S, McSweeney C, et al. 2018. Cultivation and sequencing of rumen microbiome members from the Hungate1000 Collection. Nat Biotechnol 36:359-+.
